# Health nurses’ experiences and attitudes regarding collaboration with dental personnel

**DOI:** 10.1186/s12903-016-0226-8

**Published:** 2016-06-06

**Authors:** Sonja Y. Løken, Nina J. Wang, Tove I. Wigen

**Affiliations:** Department of Paediatric Dentistry and Behavioural Science, Institute of Clinical Dentistry, University of Oslo, Box 1109, Blindern, 0317 Oslo Norway; Oral Health Centre of Expertise in Eastern Norway, Oslo, Norway

**Keywords:** Children, Dental personnel, Early childhood caries, Health nurses, Preventive oral care, Primary care personnel

## Abstract

**Background:**

Collaboration between primary care personnel and dental personnel to prevent early childhood caries has been established in several countries. The purpose of this study was, firstly, to describe health nurses’ experiences and attitudes regarding collaboration with dental personnel, and secondly, to identify characteristic of health nurses and health centres associated with the collaboration.

**Methods:**

Health nurses working with children answered a questionnaire. In total, 163 of 296 health nurses (55 %) reported demographic information, referral routines, frequency of and reasons for referral of young children to dental personnel, contact with dental personnel and satisfaction with the collaboration. Data were analysed using multivariate logistic regression.

**Results:**

The majority of health nurses (83 %) were familiar with referral routines and 31 % reported referring children to dental personnel monthly or more often. The most frequent reasons for referral were clinical caries (52 %), dental discolouration (38 %) and dental trauma (34 %). Few health nurses (18 %) had contact with dental personnel monthly or more often. Two-thirds of health nurses (71 %) reported being satisfied with the collaboration with dental personnel. Results of multivariate analysis showed that health nurses were more likely to refer children when the proportion of immigrant children under care in the health centres was high than when proportion of immigrant children was low (OR 6.4, CI 2.7–14.9). Health nurses working in small health centres were more likely to be satisfied with the collaboration than health nurses working in large health centres (OR 3.2, CI 1.4–7.0). Health nurses 45 years or older were more likely to possess knowledge of referral routines than younger health nurses (OR 2.7, CI 1.1–6.4).

**Conclusions:**

The results indicated that the majority of health nurses possessed knowledge of collaboration routines and were satisfied with the collaboration. The proportion of immigrant children under care in the health centres, the size of the health centres and the age of health nurses were factors influencing the collaboration between primary care personnel and dental personnel.

## Background

Despite the fact that early childhood caries is largely preventable, dental caries is a common chronic disease in childhood, strongly influenced by lifestyle [1, 2]. Caries prevalence among children is skewed; the majority of children have no caries and some children develop caries in the first years of life. The most recent available data from Scandinavia have shown that 5–10 % of 2- and 3-year-old children had developed caries [3, 4].

Early childhood caries and other lifestyle diseases share common risk factors and are linked to health behaviour in the family [1, 2]. Unfavourable health behaviours such as feeding and hygiene practices at an early age are documented to be associated with early caries development and may influence the child’s general health, growth and well-being [2, 5].

Collaboration between primary care personnel and dental personnel to prevent early childhood caries has been established in the US and several countries in Europe and Asia [6–8]. Dependent on country, primary care personnel could be primary care nurses, health nurses, paediatricians or general practitioners. Early intervention by health personnel has the potential to change health behaviour before diseases develop and to reduce the risk of lifestyle diseases. All children and parents have contact with health personnel during pregnancy and the child’s first years of life. The timing of a child’s first contact with dental personnel varies. While many professional associations recommend a dental visit when the first tooth erupts or by age one year [9–12], few parents bring the child to the dental services in early childhood [13]. Interprofessional collaboration, between health professionals and dental health professionals, may contribute to early identification of caries risk children and improve resource efficiency [14].

In Norway, all children are issued with an appointment for a dental examination at the age of three years. Before the first dental visit, health nurses give preventive recommendations about oral health behaviour and inspect children’s teeth at the age of two years. When visible dental plaque, clinical caries or unfavourable oral health behaviours indicating caries risk are present, children are referred to the dental services [15].

Contact between health nurses and mothers during pregnancy and infancy may facilitate early identification of children at risk of developing caries. Socioeconomic status, family relations, immigrant background and feeding habits are related to caries development in early childhood [16]. Health nurses possess knowledge of these caries risk indicators from regular contact with children and parents during the first years of life.

Collaboration between health nurses and dental personnel has been established as a consequence of national guidelines introduced in Norway ten years ago, but knowledge of the extent and details of the collaboration is scarce [17, 18]. It was hypothesized that health nurses were familiar with the referral routines, referred children for dental care and had regular contact with dental personnel.

The purpose of this study was, firstly, to describe health nurses’ experiences and attitudes regarding the collaboration with dental personnel, specifically to examine how familiar health nurses were with dental referral routines, how often they referred children for dental care, frequency of contact with dental personnel and how satisfied the health nurses were with the collaboration. Secondly, the purpose was to identify characteristic of health nurses and health centres associated with the collaboration.

## Methods

Questionnaires were mailed to all health nurses working with children in health centres in five counties in south-eastern Norway in early 2013. These counties had a population of 1.9 million, one-third of the country’s total population [19]. The questionnaires were sent by electronic mail in cooperation with local associations of health nurses. Two reminders were distributed. A total of 296 eligible health nurses were invited to participate and 163 (55 %) answered the questionnaire.

### Questionnaire

The questionnaire was partly based on questions previously tested and used on dental personnel [20–23]. Questions regarding the health nurses involvement in preventive oral care were piloted on health nurses before distribution of the questionnaire. Data were collected about characteristics of the health nurses and the health centres and about the health nurses’ experiences and attitudes regarding collaboration with dental personnel. Characteristics included the age of the health nurses, socioeconomic status of the children in the health centres' catchment area, proportion of children with immigrant background and the size of the health centres. Age of health nurses was dichotomised into less than 45 years and 45 years or older. The socioeconomic status of the children in the health centres was ranked as higher, similar or lower than the Norwegian average based on health nurses knowledge of the area. The proportion of children with immigrant background under care in the health centres was dichotomised into low (<25 %) and high (≥25 %). The size of the health centres was measured by number of children born annually in the catchment area and dichotomised into small health centres (<300 children) and large health centres (≥300 children).

Health nurses’ experiences and attitudes regarding the collaboration with dental personnel was characterised using four variables. Information was obtained by questions about frequency of contact with dental personnel, routines for referring children, frequency of referral and satisfaction with the collaboration. The frequency of contact and the frequency of referral were reported as daily, weekly, monthly, seldom and never and, in the analyses, dichotomised into monthly or more often, and more seldom than monthly. Health nurses reported whether they knew the referral routines or not. Health nurses’ satisfaction with the collaboration was measured using a five point Likert scale, ranging from very satisfied (score 1) to very dissatisfied (score 5). The scores were dichotomised into satisfied (score 1-2) or not satisfied (score 3-5) in the analyses. The health nurses reported the most frequent reasons for referring children to dental personnel. Several reasons for referring each child could be reported.

### Statistical analysis

The statistical analyses were performed using Statistical Package for the Social Sciences (SPSS, Inc. Chicago, IL, USA, version 20). Results were cross-tabulated and differences tested using Chi-square statistics. Spearman’s rank correlation was used to investigate collinearity between the independent variables before the multivariate analyses were conducted. Multivariate logistic regression analyses were conducted with the four dependent variables: frequency of contact, knowledge of referral routines, frequency of referral and the health nurses’ satisfaction with the collaboration, and the characteristics of health nurses and health centres as independent variables. Results were reported by odds ratios (OR) with 95 % confidence intervals (CI).

## Results

Table 1 shows age of the health nurses, the socioeconomic status of the children in the health centres compared with national average, proportion of children with immigrant background under care in the health centres and the size of the health centres.

**Table 1:** Characteristics of health nurses and health centres. Proportions (%) and numbers (n) of health nurses

	% (n)
Age of health nurses	
< 45 years	44 (71)
≥ 45 years	56 (92)
Children’s socioeconomic status	
High	26 (42)
Average	59 (97)
Low	15 (24)
Children with immigrant background under care in health centres	
High	29 (48)
Low	71 (115)
Size of health centres	
Large	30 (49)
Small	70 (114)

Description of the health nurses’ involvement in the collaboration with dental personnel is shown in Table 2. A minority of health nurses (18 %) reported having frequent contact with dental personnel, nearly all (83 %) reported possessing knowledge about referral routines and 31 % of the health nurses reported referring children often to dental personnel. Most health nurses (71 %) were satisfied with the collaboration.

**Table 2:** Description of the health nurses’ experiences and attitudes regarding the collaboration with dental personnel. Proportions (%) and numbers (n) of health nurses

	% (n)
Frequency of contact with dental personnel	
Monthly or more often	18 (30)
Less than monthly	82 (133)
Knowledge of referral routines	
Yes	83 (135)
No	17 (28)
Frequency of referral	
Monthly or more often	31 (51)
Less than monthly	69 (112)
Satisfaction with collaboration	
Yes	71 (116)
No	29 (47)

Table 3 shows the reasons given by health nurses for referring children to dental personnel. The most common reasons were clinical caries, tooth discolouration and dental trauma.

**Table 3:** Reasons for referring children from health nurses to dental personnel. Proportions (%) and numbers (n) of all reasons given by health nurses

	% (n)^a^
Caries risk	
Clinical caries	52 (85)
Sugary intake at night	10 (16)
Unfavourable oral behaviour	28 (45)
Parental factors	
Immigrant background	10 (17)
Parental concern	7 (11)
Dental neglect	4 (7)
Tooth factors	
Tooth eruption	6 (9)
Discolouration	38 (62)
Dental trauma	34 (56)
Miscellaneous	
No contact with dental personnel	19 (31)
Other reasons	12 (21)

^a^ Several reasons for referral could be reported

Table 4 shows the results of the bivariate analyses relating the health nurses’ experiences and attitudes regarding collaboration with dental personnel to the characteristics of health nurses and health centres. The results showed that knowledge of referral routines was associated with the age of the health nurses, frequency of referral was associated with the socioeconomic status of the children in the health centres and the proportion of children with immigrant background under care in the health centres (*p* < 0.05). The health nurses’ satisfaction with collaboration with dental personnel was associated with the size of the health centres (*p* < 0.05).

**Table 4:** Health nurses’ experiences and attitudes regarding collaboration with dental personnel to the characteristics of health nurses and health centres. Proportions (%), numbers (n) and p-values of health nurses

	Frequency of contact with dental personnel		Knowledge of referral routines		Frequency of referral to dental personnel		Satisfied with collaboration	
	Monthly or more		Yes		Monthly or more		Yes	
	% (n)	*p*	% (n)	*p*	% (n)	*p*	% (n)	*p*
Age of health nurses		ns		<0.05		ns		ns
< 45 years	40 (12)		40 (54)		41 (21)		39 (45)	
≥ 45 years	60 (18)		60 (81)		59 (30)		61 (71)	
Socioeconomic status		ns		ns		<0.05		ns
High	23 (7)		24 (33)		20 (10)		30 (35)	
Average	57 (17)		60 (81)		51 (26)		54 (63)	
Low	20 (6)		16 (21)		29 (15)		16 (18)	
Immigrants		ns		ns		<0.05		ns
Low	57 (17)		70 (95)		41 (21)		72 (84)	
High	43 (13)		30 (40)		59 (30)		28 (32)	
Size of health centres		ns		ns		ns		<0.05
Large	27 (8)		29 (39)		39 (20)		23 (27)	
Small	73 (22)		71 (96)		61 (31)		77 (89)	

Table 5 shows the results of the multivariate logistic regression analyses relating the health nurses’ experiences and attitudes regarding collaboration with dental personnel to the characteristics of health nurses and health centres. Health nurses 45 years or older were more likely to possess knowledge of referral routines than health nurses younger than 45 years of age (OR 2.7, CI 1.1–6.4). Health nurses were more likely to refer children often to dental personnel when proportion of children with immigrant background was high than when it was low (OR 6.4, CI 2.7–14.9). Health nurses were more likely to be satisfied with the collaboration with dental personnel when health centres were small than when they were large (OR 3.2, CI 1.4–7.0).

**Table 5:** Health nurses’ experiences and attitudes regarding collaboration with dental personnel to the characteristics of health nurses and health centres. Multivariate logistic regression. Odds ratios (OR) and 95 % confidence intervals (95 % CI)

	Frequency of contact with dental personnel	Knowledge of referral routines	Frequency of referral to dental personnel	Satisfied with collaboration
	Monthly or more	Yes	Monthly or more	Yes
	OR (95 % CI)	OR (95 % CI)	OR (95 % CI)	OR (95 % CI)
Age of health nurses				
< 45 years (ref)				
≥ 45 years	1.2 (0.5–2.8)	**2.7 (1.1–6.4)**	1.4 (0.6–3.0)	1.6 (0.8–3.7)
Socioeconomic status				
High (ref)				
Average	0.9 (0.4–2.5)	0.6 (0.2–1.4)	0.9 (0.3–2.2)	2.5 (1.0–6.5)
Low	0.8 (0.2–3.1)	0.4 (0.1–1.8)	0.4 (0.1–1.3)	1.1 (0.3–4.3)
Immigrants				
Low (ref)				
High	2.5 (1.0–6.4)	1.1 (0.4–3.1)	**6.4 (2.7–14.9)**	1.0 (0.4–2.4)
Size of health centres				
Large (ref)				
Small	1.7 (0.7–4.6)	1.5 (0.6–3.8)	1.1 (0.5–2.6)	**3.2 (1.4–7.0)**

Statistically significant results are marked in bold

## Discussion

The purpose of the study was to describe health nurses’ experiences and attitudes regarding the collaboration with dental personnel. The results showed that the majority of health nurses had knowledge of collaboration routines and were satisfied with the collaboration. In addition, the proportion of immigrant children under care in the health centres, size of the health centres and age of the health nurses were found to be associated with the collaboration between health nurses and dental personnel.

This study was a questionnaire study. Although limitations such as non-responses, misconceptions and errors are present in all questionnaire studies, the probability of recall and report error in the present study were considered limited as most questions were related to daily work of health nurses [24]. The response rate was somewhat lower than in similar studies including dental personnel [18]. This may reflect the fact that oral care is one of many duties for health nurses, while oral health is the main focus for dental personnel. Income, expenditure and educational level in the studied area did not differ from the Norwegian average, suggesting that the results were representative for the country in general [19].

### Knowledge of referral routines

Most health nurses had knowledge of existing routines for referring children to dental personnel. This indicated that routines were established and implemented in the health centres. Health nurses have reported dental personnel to be the most important source of knowledge about oral health [18]. The study showed that health nurses 45 years and older were more likely to possess knowledge of referral routines than younger health nurses. It is reasonable to assume that knowledge and professional skills gradually accumulate by age. Another explanation may be that health nurses with long clinical experience have established personal contact with dental personnel. This may indicate that internal communication in health centres’ between older, experienced and younger health nurses could be improved.

### Reasons for referring children

The most frequent reason for referring children to dental personnel was clinical caries. The present study showed that health nurses identified children at risk of developing caries, though one study has shown that the curriculum in oral health in health nurses’ education was random and limited [17].

### Frequency of referral

The majority of health nurses reported having referred children to dental personnel less frequently than monthly. One explanation may be that caries prevalence among young children was low (7 % of 2 year olds) in the studied area [16, 25]. Other explanations may be that health nurses lacked sufficient knowledge to identify caries risk children or that they did not prioritise oral health. The possibility that some health nurses under-referred children in need of treatment cannot be excluded. One study has reported that 30 % of preschool children with signs of dental caries were not referred by primary care personnel [26].

The frequency of referral to dental personnel was strongly related to the proportion of children with immigrant background in health centres. It is well documented that immigrant children develop caries more often than other children [16, 27]. The results from the present study suggest that health nurses were aware of immigrant background as a risk factor for early childhood caries and that they were focused on the identification of caries in immigrant children.

### Satisfaction with collaboration

Most health nurses were satisfied with the collaboration with dental personnel. This may indicate that health nurses had been given sufficient information from dental personnel to provide oral health promotion and to identify children at risk of developing caries. It has been reported that primary care personnel considered that to identify oral health problems and provide preventive oral care to children and parents were important parts of their duties [28]. Health nurses working in small health centres were more likely to be satisfied with the collaboration than health nurses working in large health centres. In small health centres, health nurses have the opportunity for building a close relationship with children, parents and dental personnel and the overall responsibility for the child population, from pregnancy to school age.

### Frequency of contact

A minority of health nurses reported having frequent personal contact with dental personnel. In Norway, dental hygienists organise meetings with health nurses, usually once a year. Although this study showed that few health nurses had frequent personal contact with dental personnel, the majority were satisfied with the collaboration. This may indicate that yearly meetings were sufficient to keep the health nurses updated and motivated to maintain the caries preventive focus.

### Interprofessional collaboration

The common risk approach provides a rationale for integrating oral health promotion into general health messages, which may facilitate early identification of risk children by interprofessional collaboration [1, 2]. In low caries populations, where few children need oral health intervention in the first years of life, collaboration with primary care personnel may be cost-effective. Scheduled visits at health centres for infants and toddlers are established in Norway. Interprofessional collaboration has been shown to improve the consistency of oral health promotion given to children and parents by different health professions [18].

## Conclusions

This study indicated that the majority of health nurses possessed knowledge of collaboration routines and were satisfied with the collaboration. The proportion of immigrant children under care in the health centres, the size of the health centres and the age of health nurses were characteristics associated with the collaboration between health nurses and dental personnel. As health nurses were satisfied with the collaboration, the results may suggest that they felt able to identify oral problems and provide oral health promotion for young children. Further studies are needed to determine whether all children in need of dental care are being referred to dental personnel by health nurses.

## Abbreviations

None
